# Poveri! Poveris!

**Published:** 1889-01

**Authors:** Joaquin Miller


### "POVERI! POVERIS!"

"Feed my sheep."

Come, let us ponder ; it is fit—
  Born of the poor, born to the poor.
The poor of purse, the poor of wit,
  Were first to find God's opened door—
Were first to climb the ladder round by round
That fell from heaven's door unto the ground.

God's poor came first, the very first !
  God's poor were first to see, to hear,
To feel the light of heaven burst
  Full on their faces. Far or near,
His poor were first to follow, first to fall !
What if at last his poor stand first of all ?

Joaquin Miller, in the November *Century*.

